# Diagnosis of Swine Toxoplasmosis by PCR and Genotyping of *Toxoplasma gondii* from pigs in Henan, Central China

**DOI:** 10.1186/s12917-017-1079-3

**Published:** 2017-05-31

**Authors:** Haiyan Wang, Longxian Zhang, Qinge Ren, Fuchang Yu, Yurong Yang

**Affiliations:** 1 College of Animal Science and Veterinary Medicine, Henan Agricultural University, Zhengzhou, 450002 Henan People’s Republic of China; 2 Department of Animal Science, Henan Vocational College of Agriculture, Zhongmu, 451450 Henan, People’s Republic of China

**Keywords:** Swine, Toxoplasmosis, PCR assay, Diagnosis, Molecular characterization, Henan

## Abstract

**Background:**

*Toxoplasma gondii,* a widely prevalent protozoan parasite, causes serious toxoplasmosis infections in humans and other animals. Among livestock, pigs are susceptible to *T. gondii* infection. Despite Henan being one of the biggest pig-raising provinces in China, little information exists on the epidemiology of toxoplasmosis in this location. Therefore, we molecularly characterized DNA samples from pigs in Henan. A total of 1647 samples, including 952 from dead piglets, 478 from seriously sick fattening pigs and 217 from abortion sows, were collected from different animal hospitals or pig farms from 10 different cities in Henan (2006–2008). Each pig corresponded to a separate pig farm. DNA was extracted from 3 to 5 g of the most severely affected pig tissue (liver, spleen, lung, hilar lymph nodes and amniotic fluid) after postmortem examination. The presence of the *T. gondii* B1 gene was detected using nested polymerase chain reactions (PCR). Genotyping was performed directly on DNA from the PCR-positive tissue samples using 11 PCR restriction fragment length polymorphism markers (SAG1, 5′- and 3′-SAG2, alternative SAG2, SAG3, BTUB, GRA6, L358, PK1, c22–8, c29–2, and Apico).

**Results:**

Of all samples, thirty-four were positive for the *T. gondii* B1 gene (2.06%, 95% CI: 1.86%–2.26%) from four cities, including 31 from NanYang city, one (PgXY 1) from Xinyang City, one (PgZZ 1) from Zhengzhou City and one (PgZK1) from Zhoukou City. The prevalence was found to be highest in piglets than in fattening pigs and sows. And the difference was statistically significant (P<0.01). The following 32 samples were genotyped with complete data: 13 hilar lymph node tissue samples, seven liver tissue samples, seven lung tissue samples, four spleen tissue samples, and one amniotic fluid sample. Only one genotype, belonging to ToxoDB Genotype #9, was identified.

**Conclusions:**

This is the first large-scale survey molecularly characterizing *T. gondii* from pigs in Henan. The results of the present study revealed that *T. gondii* infection is present in swine in Henan and is a potential source of foodborne toxoplasmosis in the investigated areas. Implementation of effective control measures for *T. gondii* to reduce the chance of zoonotic toxoplasmosis spreading from pig farms may be warranted. The results show that the ToxoDB #9 genotype may be the dominant *T. gondii* lineage in mainland China. These findings strengthen the limited Chinese *T. gondii* epidemiology database.

## Background

*Toxoplasma gondii,* a common worldwide zoonotic protozoan parasite, infects a large number of warm blooded vertebrates. Humans become infected postnatally mainly by ingesting infectious oocysts from the environment or by consumption of undercooked or raw meat that contains tissue cysts. Although *T. gondii* generally causes a mild and self-limited infection in immunocompetent individuals, it can cause abortion, fetal abnormalities and prenatal death in pregnant women, and can be fatal to some immunocompromised patients such as those with AIDS or organ transplant recipients [1]. *T. gondii* infections in humans have recently been shown to be associated with an increased risk of schizophrenia [2]. It has been estimated that 33% of the world’s population and 7.9% of the population in China have been exposed to this parasite [3, 4].

Among livestock, pigs are susceptible to infection with *T. gondii* and infected pigs are considered to be one of the most important sources of *T. gondii* infection in humans [5]. Therefore, the potential risk of transmission of the disease to humans by consumption of undercooked or raw pork is an important food safety issue.

Efforts have been made to genetically compare *T. gondii* strains from different animals with those from humans to reveal the transmission dynamics of the parasite and to gain better control of its spread. Based on multilocus restriction length polymorphism analysis (RFLP) or multilocus enzyme electrophoresis, three clonal lineages (type I, II and III) are predominant in Europe and North America [6]. However, recently, a few genetically diverse *T. gondii* isolates, designated as ‘atypical’ or ‘exotic’ have been found circulating in animals and humans in other geographical regions [7–9].

Despite the large literary data on toxoplasmosis worldwide, studies on the occurrence and genetic characterization of *T. gondii* isolates from pigs in China are rather scarce [10–16], although pork is one of the most popular meats, representing around 65% of meat consumption in this country [14]. Henan, central China, is one of the biggest producers of pork products in China. About 105 million pigs were reared in Henan in 2015, making Henan the country’s second largest pork producer. However, information on toxoplasma in pigs is limited in Henan. Thus, the aim of this study was to diagnose swine toxoplasmosis in Henan by molecular methodology and to genetically characterize the causative agent of this serious parasitic zoonosis. The data obtained will augment the currently limited epidemiological database on *T. gondii* in China.

## Methods

### Collection area

**Fig. 1:**
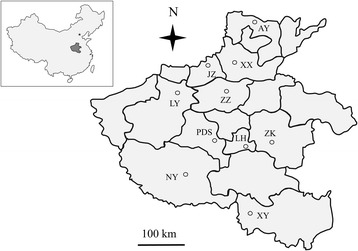
Map showing Henan(HN), central China, where pigs from different animal hospitals or farms were sampled from Anyang City (AY), Xinxiang City (XX), Jiaozuo City (JZ), Luoyang City (LY), Zhengzhou City (ZZ), Pingdingshan City (PDS), Luohe City (LH), Zhoukou City (ZK), Nanyang City (NY) and Xinyang City (XY). The shadowed area of Henan is enlarged for clarity. The figure was generated using the softwares of MapInfo Professional 7.0, Microsoft PowerPoint 2007 and Adobe Photoshop 13.0

Henan, which lies between latitude 31°23′–36°22′ degrees north and longitude 110°21′–116°39 degrees east, is also known as central China. The province is about 550 km from north to south, and about 580 km from east to west (Fig. 1). It has a distinct temperate continental monsoon climate, with four clearly delineated seasons. The average annual temperature of the province is 12–16 °C, with the lowest temperature occurring in January (−5–3 °C) and the highest temperature occurring in July (28–33 °C).

### Sample collection

During the period from September 2006 to December 2008, after permission was obtained from the animal hospitals and the pig farm owners, a total of 1647 sick or dead pigs, including 952 dead piglets, 478 seriously sick fattening pigs and 217 abortion sows, were collected from different animal hospitals or pig farms from 10 different cities in Henan (Table 1). Each pig corresponds to a pig farm, including small peasant households and small- or large-scale intensive breeding farms. The animals were free-range or from semi-intensive rearing systems in rural areas. For each animal, 3–5 g of the most seriously affected pathological tissues (liver, spleen, lung, hilar lymph nodes and amniotic fluid), as determined by postmortem examination (subcutaneous hemorrhage, enlargement and necrosis), were collected. Freshly collected samples were placed into clean plastic bags and transported immediately to the laboratory where they were stored at −20 °C. The date of collection, age, and farm of origin for each animal was recorded in detail.

**Table 1:** Prevalence of *T. gondii* by PCR in different cities in Henan Province, China

Location	No. positive/No. examined (%)			
	Piglets	Fattening pigs	Sows	Total
Zhengzhou	1/132(0.76)	0/86(0)	0/72(0)	1/290(0.34)
Nanyang	29/185(15.7)	0/102(0)	2/85(2.36)	31/372(8.33)
Xinxiang	0/98(0)	0/31(0)	0/7(0)	0/136(0)
Luohe	0/64(0)	0/38(0)	0/16(0)	0/118(0)
Jiaozuo	0/65(0)	0/20(0)	0/0(0)	0/85(0)
Xinyang	1/73(1.37)	0/95(0)	0/23(0)	1/191(0.52)
Luoyang	0/75(0)	0/35(0)	0/0(0)	0/110(0)
Pingdingshan	0/80(0)	0/28(0)	0/2(0)	0/110(0)
Anyang	0/98(0)	0/25(0)	0/0(0)	0/123(0)
Zhoukou	1/82(0)	0/18(0)	0/12(0)	1/112(0.89)
Total	32/952(3.36)	0/478(0)	2/217(0.92)	34/1647(2.06)

### DNA extraction

The freshly collected samples were grinded up and digested in a trypsin solution for 2 h at 37 °C, and the pellets were stored at −20 °C until use. Genomic DNA was extracted from the processed tissues using a tissue Gen DNA kit (CWBIO, Beijing, China), according to the manufacturer’s recommendations. The DNA samples were prepared after purification by silica gel column chromatography, eluted into 50 μL of elution buffer and stored at −20 °C until use.

### PCR analysis

A nested PCR targeting the *T. gondii* B1 gene was performed to detect possible infection with *T. gondii* [17]. A 194 bp fragment was PCR amplified using two primer sets. The first fragment was amplified with the F1 (5′-TCTTTAAAGCGTTCGTGGTC-3′) and R1 (5′-GGAACTGCATCCGTTCATGAG-3′) primer set, while the second fragment was amplified with the F2 (5′-GGCGACCAATCTGCGAATACACC-3′) and R2 (5′-TGCATAGGTTGCAGTCACTG-3′) primer set. The PCRs were conducted in 25 μL volumes containing 1 × PCR buffer (Takara Shuzo Co., Ltd., Otsu, Japan), 1.5 mM MgCl_2_, 0.2 mM of each dNTP (Takara Shuzo Co., Ltd), 0.4 μM of each primer, 1 unit of rTaq DNA polymerase (Takara Shuzo Co., Ltd.), and 2 μL of each individual DNA sample. The first amplification consisted of an initial denaturation step of 10 min at 94 °C, followed by 35 cycles of 10 s at 93 °C, 10 s at 62.5 °C and 15 s at 72 °C, and a final extension step of 10 min at 72 °C. The second amplification consisted of an initial denaturation step of 10 min at 94 °C, followed by 35 cycles of 10 s at 93 °C, 10 s at 57 °C and 30 s at 72 °C, and a final extension step of 10 min at 72 °C. Positive (RH strain) and negative (distilled water) control samples were included in each PCR run. The amplification products were electrophoretically separated on 1.5% agarose gels containing ethidium bromide and then visualized on a UV transilluminator.

### Multilocus PCR-RFLP

Genetic characterization of all the *T. gondii-*positive samples was conducted using the multilocus PCR-RFLP method described by Su et al. [18]. In brief, the target DNA sequences were amplified by multiplex PCR using external primers for 11 genetic markers: SAG1, 5′ - and 3′ -SAG2, alternative SAG2, SAG3, BTUB, GRA6, L358, PK1, c22–8, c29–2, and Apico. The multiplex PCRs were each carried out in a 25 μL volume containing 1 × PCR buffer, 2 mM MgCl_2_, 200 μM of each dNTP, 0.15 μM each of the external forward and reverse primers, 1 unit of FastStart DNA polymerase (Takara Shuzo Co., Ltd) and 1.5 μL of each DNA sample. The reaction mixture was heated to 95 °C for 4 min, followed by 30 cycles of 94 °C for 30 s, 55 °C for 1 min and 72 °C for 2 min. The multiplex PCR amplified products were diluted 1:1 by adding 25 μL of nuclease-free water. Then, 1 μL of each product served as the template DNA for nested PCR using the internal primers for each marker. The reaction mixture was heated at 95 °C for 4 min, followed by 35 cycles of 94 °C for 30 s, 60 °C for 1 min and 72 °C for 1.5 min (with an annealing temperature of 58 °C instead of 60 °C for the Apico marker). Each nested PCR product sample (5 μL) was digested with restriction endonucleases in a 20 μL volume and then resolved by 3% agarose gel electrophoresis to reveal the DNA banding pattern. The banding patterns of the samples were compared with the genotypes deposited in ToxoDB (http://toxodb.org/toxo/) to determine the *T. gondii* genotypes.

## Results

In total, of the 1647 DNA samples we analyzed, 34 (2.06%, 95% CI: 1.86%–2.26%) from four cities were positive for the *T. gondii* B1 gene. These comprised 31 (PgNY 1–31) from Nanyang City, one (PgXY 1) from Xinyang City, one (PgZZ 1) from Zhengzhou City and one (PgZK1) from Zhoukou City. Nanyang City had the majority of *T. gondii*-positive farms. The prevalence was found to be highest in piglets than in fattening pigs and sows (P<0.01) (Table 1).

All the *T. gondii*-positive samples were identified by multilocus PCR-RFLP. The low DNA concentrations of the samples meant that only 32 of them generated complete genotyping results from the 11 PCR-RFLP markers. Two samples (PgNY 6 and 19) were genotyped at nine or fewer genetic loci. It is interesting that only one genotype (ToxoDB#9) from all the *T. gondii* samples from different cities in Henan was identified, while typical type I, II and III lineages, which are predominant in North America and Europe, were not found in our study. Table 2 shows the genotyping results for these samples and the ToxoD database reference information.

**Table 2:** Multilocus genotyping of *T. gondii* isolates from different porcine hosts and geographical locations worldwide by PCR-RFLP

Isolate ID	Host	Tissue	Location	Age	PCR RFLP genotype (genetic marker)											
					SAG1	5′ + 3′ SAG2	Alt. SAG2	SAG3	BTUB	GRA6	C22–8	C29–2	L358	PK1	Apico	Genotype
RH	Human		USA		I	I	I	I	I	I	I	I	I	I	I	Reference, ToxoDB #10 (type I)
PIH	Human		USA		II	II	II	II	II	II	II	II	II	II	II	Reference, ToxoDB #1 (type II)
CTG	Cat		USA		III	III	III	III	III	III	III	III	III	III	III	Reference, ToxoDB #2 (type III)
MAS	Human		France		u-1	I	II	III	III	III	u-1	I	I	III	I	Reference, ToxoDB #17
TgCgCa1	Cougar		Canada		I	I	II	III	III	II	II	u-1	I	u-2	I	Reference, ToxoDB #66
TgCatBr5	Cat		Brazil		I	III	III	III	II	III	I	I	I	u-1	I	Reference, ToxoDB #19
PgNY 1–5 (*n* = 5)	Pig	Liver	Ny, Henan	10–15 days	u-1	II	II	III	III	II	II	III	II	II	I	ToxoDB #9
PgNY 6	Pig	Liver	Ny, Henan	1–7 days	u-1	II	II	III	III	II	nd	III	II	nd	I	ToxoDB #9?
PgNY 20–31 (*n* = 12)	Pig	Hld	Ny, Henan	10–15 days	u-1	II	II	III	III	II	II	III	II	II	I	ToxoDB #9
PgNY 7–10,14 (*n* = 5)	Pig	Lung	Ny, Henan	1–7 days	u-1	II	II	III	III	II	II	III	II	II	I	ToxoDB #9
PgNY 11,16,17 (*n* = 3)	Pig	Spleen	Ny, Henan	20–30 days	u-1	II	II	III	III	II	II	III	II	II	I	ToxoDB #9
PgNY 12,15 (*n* = 2)	Pig	Lung	Ny, Henan	1–2 months	u-1	II	II	III	III	II	II	III	II	II	I	ToxoDB #9
PgNY 13,18 (*n* = 2)	Pig	Af	Ny, Henan	Sow	u-1	II	II	III	III	II	II	III	II	II	I	ToxoDB #9
PgNY 19	Pig	Spleen	Ny, Henan	20–30 days	u-1	nd	nd	III	III	II	II	III	II	nd	I	ToxoDB #9?
PgZK 1	Pig	Liver	Zk, Henan	20–30 days	u-1	II	II	III	III	II	II	III	II	II	I	ToxoDB #9
PgXX 1	Pig	Liver	Xy, Henan	1–2 months	u-1	II	II	III	III	II	II	III	II	II	I	ToxoDB #9
PgZZ 1	Pig	Hld	Zz, Henan	1–7 days	u-1	II	II	III	III	II	II	III	II	II	I	ToxoDB #9

nd, no data. u-1 and u-2 are unique RFLP genotypes

Hld and Af denote hilar lymph node and amniotic fluid, respectively

Ny, Zk, Xy and Zz denote the cities of Nanyang, Zhoukou, Xinyang and Zhengzhou, respectively

## Discussion

Infection with *T. gondii* is relatively common in pigs. The prevalence of *T. gondii* differs greatly among countries and regions within the same country. In the present study, the prevalence of *T. gondii* was 2.06% in pigs, a value lower than those reported in other studies in China [11–13]. Because of the lack of existing epidemiological data on swine toxoplasmosis in the areas we investigated, we cannot speculate as to why a low toxoplasma prevalence rate was obtained. Prevalence rates can be affected by the detection methods used, the tissue type sampled, sample sizes, animal age differences, and animal housing conditions, along with many other environmental and farm management factors. The prevalence rate was higher in Nanyang than in the other areas we tested, which could be related to Nanyang being the poorest city of those we investigated. Although it possesses a substantial pig population, this population is dominated by small peasant households that breed pigs in resource limited environments with poor animal management. The data from this region is similar to that in a Latvia report where the prevalence of *T. gondii*-specific antibodies was found to be greater in free-ranging domestic pigs (6.2%, *P* < 0.05) than in intensively farmed pigs (0.4%) [19]. Also, once *T. gondii* cysts have formed in the brain and muscles, toxoplasmosis does not usually show any clinical signs or visible damage in immune competent animals, a situation that may lead to underestimates of the disease prevalence. Indeed, a study from Anhui province, eastern China, found that a significant proportion (18.03%) of commercially available pork is contaminated with *T. gondii* based on the real-time PCR results [14].

In the present study, the prevalence of *T. gondii* was notably high in piglets. This might be explained by piglets being more susceptible to all kinds of opportunistic organisms, including *T. gondii*, and clinical manifestations may appear when their immunity declines under the poor animal management and the presence of some immune system diseases (e.g. porcine circovirus or porcine reproductive and respiratory syndrome).

Our data show that swine toxoplasmosis exists in pig farms in Henan Province. Incorrect diagnosis or poor management of the disease may cause wider economic losses for swine herd production. Importantly, some asymptomatic animals with *T. gondii* cysts in their brains and muscle tissues may circulate in the fresh pork market and become a source of foodborne toxoplasmosis, thus posing a food safety threat to public health. Therefore, integrated control strategies and measures should be implemented on farms to prevent *T. gondii* infection and reduce the risk of spreading zoonotic toxoplasmosis.

The differential virulence of *T. gondii* strains has made the study of genetic diversity in *T. gondii* isolates an interesting and important research topic. Three predominant clonal types (i.e., I, II, and III) are commonly described in the scientific literature. Type I strains are highly virulent in mice, whereas type II and III strains are avirulent or non virulent [6]. Here, only one genotype (ToxoDB#9) was identified, while typical type I, II and III lineages, which have been predominantly described in pigs in North America and Europe [6], were not found in our study. This result is similar to the results reported by other authors in studies of *T. gondii* parasites isolated from pigs, yaks, cats, rats, mice, *Microtus fortis*, waterfowl and humans in eastern China (Anhui and Jiangsu) [14, 20, 21], southern China (Guangdong, Yunnan) [10, 22, 23], central China (Henan, Hubei and Jiangxi) [10, 11, 24–27], northern China (Beijing and Jilin) [28–30] and western China (Gansu, Yunnan and Guizhou) [12, 15, 31–35]. All current and past RFLP genotyping data for *T. gondii* isolates from China are summarized in Table 3. The compiled results provide solid evidence that genotype ToxoDB#9 has been, and remains, the predominant lineage in mainland China. Genotype ToxoDB#9 may also have a more worldwide distribution, circulating in South and North America, as well as in eastern Asia.

**Table 3:** Genetic PCR-RFLP *T. gondii* types in animals and humans in China

Host	No. of isolates	PCR RFLP genotype												References
		#10 (Type I)	#1 (Type II)	#2 (Type III)	#3 (Type II variant)	#3	#9	#18	#204	#205	#225	#213	#20	
Pig	65	15	0	0	1	4	43	0	0	0	0	2	0	[10, 11, 13–15, 25, 31, 36]
Cat	96	1	2	0	1	21	62	2	0	4	2	0	1	[30–33, 10, 15, 22–24, 28, 35]
yaks	2	0	0	0	0	0	2	0	0	0	0	0	0	[34]
Bird	6	0	0	0	4	0	2	0	0	0	0	0	0	[37]
Human	15	2	1	0	0	1	10	0	1	0	0	0	0	[10, 21, 32]
Sheep	1	0	1	0	0	0	0	0	0	0	0	0	0	[10]
Rat	4	0	0	0	0	0	4	0	0	0	0	0	0	[16]
Mouse	3	0	0	0	0	0	3	0	0	0	0	0	0	[16]
Goat	8	7	0	0	0	0	1	0	0	0	0	0	0	[36]
Vole	1	0	0	0	0	0	1	0	0	0	0	0	0	[31]
Chicken	1	0	0	0	0	0	0	0	0	0	1	0	0	[31]
Deer	6	0	0	0	0	0	6	0	0	0	0	0	0	[38]
Fox	5	2	0	0	0	0	3	0	0	0	0	0	0	[39]
Rabbit	1	0	0	1	0	0	0	0	0	0	0	0	0	[40]
Total	214	27 (12.6%)	4 (1.9%)	1 (0.5%)	6 (2.8%)	26 (12.1%)	137 (64.0%)	2 (0.9%)	1 (0.5%)	4 (1.9%)	3 (1.4%)	2 (0.9%)	1 (0.5%)	

## Conclusions

This is the first large-scale study to molecularly characterize *T. gondii* from pigs in central China. Our results have revealed that *T. gondii* infection is present in swine in Henan and may, therefore, pose a threat to public health via the consumption of raw or undercooked infected pork from this location. PCR-RFLP identified only one genotype (ToxoDB #9) in Henan. These findings augment the limited *T. gondii* epidemiology database in China. To better understand the molecular epidemiology and population structure of *T. gondii* in Henan, it will be necessary to sample a wider variety of animal hosts.

## Abbreviations

RFLP: Restriction fragment length polymorphism
Hld: Hilar lymph node
Af: Amniotic fluid
nd: no data
